# The impairment of intramural periarterial drainage in brain after subarachnoid hemorrhage

**DOI:** 10.1186/s40478-022-01492-8

**Published:** 2022-12-18

**Authors:** Yanrong Sun, E. Liu, Yanhong Pei, Qinhan Yao, Haowen Ma, Yakun Mu, Yingjie Wang, Yan Zhang, Xiaomei Yang, Xing Wang, Jiajia Xue, Jiliang Zhai, Roxana O. Carare, Lihua Qin, Junhao Yan

**Affiliations:** 1 Department of Anatomy, Histology and Embryology, School of Basic Medical Sciences, Peking University Health Science Center, Beijing, 100191 China; 2 Department of Anatomy, School of Medicine, Shandong University, Jinan, 250012 Shandong China; 3 State Key Laboratory of Organic-Inorganic Composites, Beijing Laboratory of Biomedical Materials, Beijing University of Chemical Technology, Beijing, 100029 China; 4 Department of Orthopaedic Surgery, Peking Union Medical College Hospital, Chinese Academy of Medical Science and Peking Union Medical College, Beijing, 100730 China; 5 Faculty of Medicine, UK Southampton General Hospital, University of Southampton, Southampton, SO16 6YD UK; 6 University of Medicine, Pharmacy, Science and Technology “G.E. Palade”, Targu Mures, Romania; 7 Beijing Key Lab of Magnetic Resonance Imaging Technology, Peking University Third Hospital, Beijing, 100191 China

**Keywords:** Intramural periarterial drainage, Subarachnoid hemorrhage, Matrix metalloproteinase 9, Collagen type IV, Interstitial fluid, Cerebrospinal fluid

## Abstract

Interstitial fluid (ISF) from brain drains along the basement membranes of capillaries and arteries as Intramural Periarterial Drainage (IPAD); failure of IPAD results in cerebral amyloid angiopathy (CAA). In this study, we test the hypothesis that IPAD fails after subarachnoid haemorrhage (SAH). The rat SAH model was established using endovascular perforation method. Fluorescence dyes with various molecular weights were injected into cisterna magna of rats, and the pattern of IPAD after SAH was detected using immunofluorescence staining, two-photon fluorescent microscope, transmission electron microscope and magnetic resonance imaging tracking techniques. Our results showed that fluorescence dyes entered the brain along a periarterial compartment and were cleared from brain along the basement membranes of the capillaries, with different patterns based on individual molecular weights. After SAH, there was significant impairment in the IPAD system: marked expansion of perivascular spaces, and ISF clearance rate was significantly decreased, associated with the apoptosis of endothelial cells, activation of astrocytes, over-expression of matrix metalloproteinase 9 and loss of collagen type IV. In conclusion, experimental SAH leads to a failure of IPAD, clinically significant for long term complications such as CAA, following SAH.

## Introduction

The cerebrospinal fluid (CSF) in the subarachnoid space flows into interstitial spaces (ISS) along the pial-glial basement membranes also named perivascular spaces (PVS) and mixes with the interstitial fluid (ISF) [1]. Based on the glymphatic system theory, the ISF is then cleared from the brain parenchyma along the para-venous route facilitated by aquaporin-4 (AQP4) expressed by astrocytes [6, 18, 25]. It is controversial how much the para-venous pathway is involved in the efflux of soluble substances in ISS and this process cannot provide an explanation for cerebral amyloid angiopathy (CAA) that occurs mainly in the walls of capillaries and arteries [9]. Intramural periarterial drainage (IPAD) occurs along the basement membranes of capillaries and the basement membranes surrounding smooth muscle cells of cerebral arteries. The motive force for IPAD is provided by the spontaneous contractions of smooth muscle cells and IPAD fails with ageing [3, 16], resulting in CAA.

IPAD is impaired in Alzheimer’s disease (AD) and stroke [11, 20]. Subarachnoid hemorrhage (SAH) is a type of hemorrhagic stroke mainly caused by rupture of intracranial aneurysms. After SAH, the blood cell lysates in the CSF (such as the macro-molecule methemoglobin and micro-molecule heme) flow into the ISS through PVS. These lysates trigger inflammatory reactions and apoptotic processes, resulting in neuro-vascular unit injury, such as disruption of blood brain-barrier (BBB), apoptosis of endothelial cells and neurons, brain edema and cerebral vasospasm [13, 29].

Matrix metalloproteinases (MMPs) comprises a large family of zinc endopeptisases that collectively modify almost all components of the extracellular matrix in the brain. MMP9 is upregulated in human stroke and many animal models of cerebral ischemia, intracerebral hemorrhage and subarachnoid hemorrhage [17, 27]. The activation of MMP9 has been associated with degradation of vascular basement membranes (e.g. collagen type IV, Col-IV) and the development of vasogenic edema after transient focal ischemia [24].

In this study, using a model of SAH, we demonstrated apoptosis of endothelial cells, activation of astrocytes, degradation of capillary basement membranes and impairment of IPAD after SAH.

## Materials and methods

This study was performed according to the national guidelines for the use of experimental animals, and the study protocols had been approved by the Ethics Committee of Peking University Health Center. The “principles of laboratory animal care” (NIH publication No. 86–23, revised 1985) were strictly followed in this study.

### Study design

**Fig. 1:**
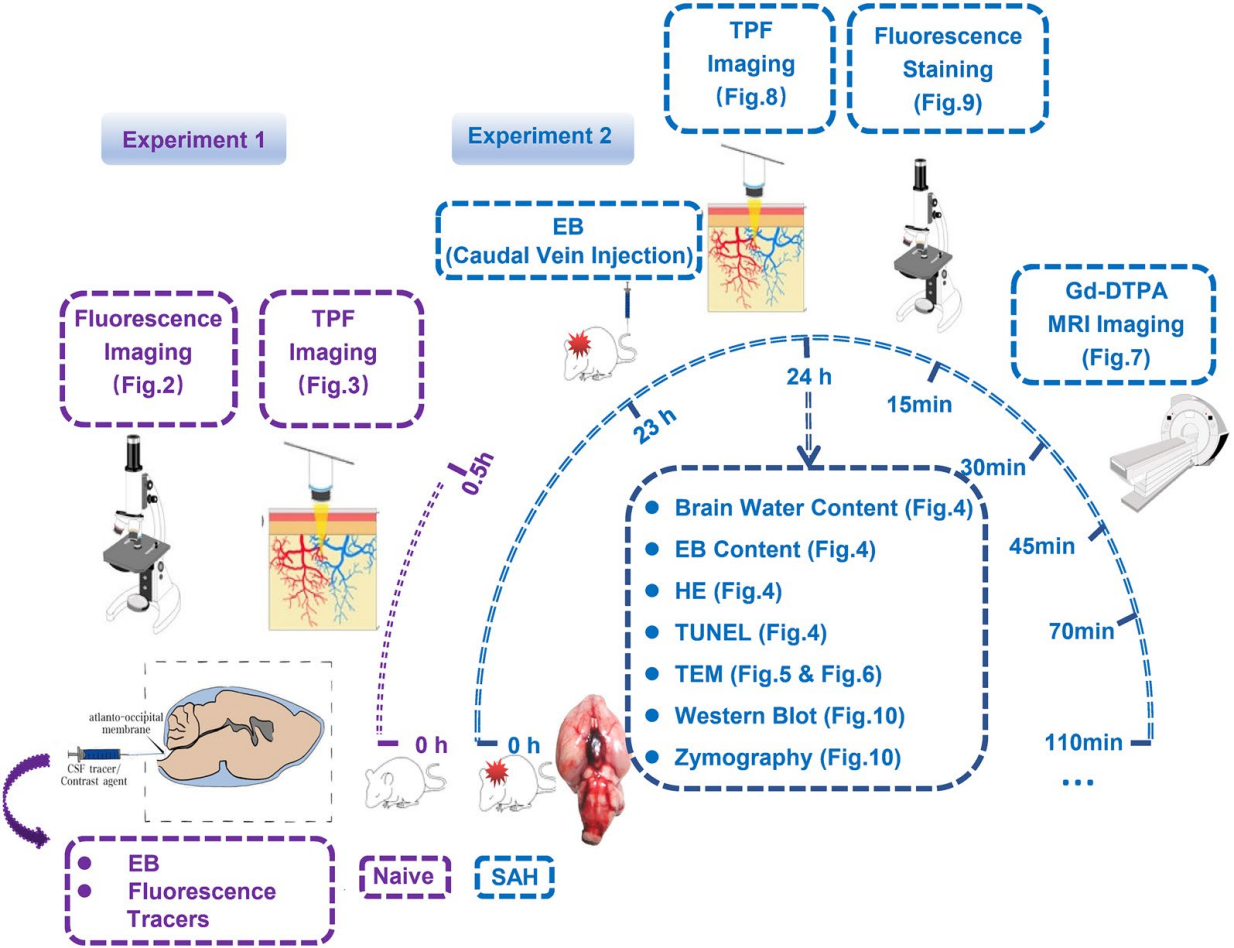
The protocol of the study. In Experiment 1, the fluorescence tracers with different molecular weights and EB dye were injected into the cisterna magna of naive rats, and at 0.5 h after injection, the individual distribution patterns of these fluorescence tracers were explored using TPF imaging. The distribution pattern of EB was observed before and after dcLN ligation. In Experiment 2, the pattern of IPAD was analysed at 24 h after establishment of SAH model. TPF imaging was applied to explore the distribution changes of these fluorescence tracers, and the ISF flow alteration after SAH was analyzed using Gd-DTPA MRI technique. The disruption of BBB, apoptosis of endothelial cells and basement membrane degradation were revealed using EB fluorescence imaging, TUNEL, MMP9 and IV-Col staining and MMP9 zymography. Three rats were randomly selected in each group involved in each item of experiment 1, and 5 rats were randomly selected in each group involved in each item of experiment 2. Abbreviations: dcLN, deep cervical lymph node; EB, Evans Blue; Gd-DTPA, gadolinium-diethylenetriaminepentaacetic acid; IPAD, intramural periarterial drainage; ISF, interstitial fluid; IV-Col, collage type IV; MMP9, matrix metalloproteinase 9; MRI, magnetic resonance imaging; SAH, subarachnoid hemorrhage; TEM, transmission electron microscopy; TPF, two-photon fluorescence; TUNEL, TdT-mediated dUTP Nick-End Labeling; WB, western blot

The study was divided into two experiments. In the Experiment 1, Evans Blue (EB, 10 kD) was injected into the cisterna magna, and the distribution pattern of EB was detected by fluorescence imaging before and after ligation of deep cervical lymph nodes (dcLN). Fluorescent tracers with different molecular weights and EB dye were injected into the cisterna magna of Naive rats, and at 0.5 h after injection; two-photon fluorescent (TPF) imaging was performed to explore the individual distribution patterns of these fluorescence tracers. In Experiment 2, at 24 h after establishment of SAH model, the water content and EB content in brain, the expansion and ultrastructural changes of IPAD were analyzed. As in Experiment 1, TPF imaging was also used to explore the distribution changes of the fluorescent tracers and the ISF flow alteration after SAH was also analyzed using gadolinium-diethylenetriaminepentaacetic acid (Gd-DTPA) MRI technique. The disruption of BBB and apoptosis of endothelial cells were studied using EB fluorescence imaging, TdT-mediated dUTP Nick-End Labeling (TUNEL), MMP9 and IV-Col staining and MMP9 zymography. Details are shown in Fig. 1.

### Animal model

In Experiment 1, the 10 male Sprague-Dawley rats were randomly divided into Naive group (n = 6) and dcLN ligation group (n = 3), and 1 rat died after dcLN ligation. In Experiment 2, 89 rats were divided into Sham and SAH groups (n = 35 each group), and 19 rats died after SAH. The rats were housed with 12 h light / dark cycles and controlled temperature (22 ± 1 °C) and humidity (60% ± 5%).

Three rats were randomly selected from Naive group and dcLN ligation group, respectively (n = 3). The rat dcLN ligation model was performed as previously described [31]. Animals were anesthetized by 5% isoflurane and ventilated with 3% isoflurane (65% medical air with 35% oxygen gas) during surgery. A longitudinal incision was made on the neck from the mandible to the sternum. The muscles and fascia were separated along the trachea using a tweezer. The dcLNs were identified below the sternothyroid muscle and the afferent and efferent vessels were ligated using nylon suture (6–0). Sham-operated rats were only exposed dcLNs without ligation.

The rat SAH model was established using a modified endovascular perforation method as previously described [35]. Briefly, the animal was anesthetized as above during surgery. A midline incision was made in the neck, a 3 cm sharpened monofilament nylon suture (4–0) was introduced into the external carotid artery, advanced to the bifurcation of the anterior and middle cerebral arteries, and further inserted slightly (about 3 mm). Then, the nylon suture was withdrawn immediately. The animals in Sham group underwent the same surgery procedure without artery perforation.

### Measurement of brain water content

Five rats were randomly selected from Sham group and SAH group respectively (n = 5), and the brain water content of each group was assessed as previously reported [38]. At 24 h after SAH, the rats were anesthetized as above, and the whole brain was harvested and weighed immediately (wet weight), and weighed again after drying in an oven at 105 °C for 24 h (dry weight). The brain water content was calculated as: [(wet weight-dry weight) / wet weight] × 100%.

### ISF tracking

Three rats were randomly selected from Naive group (n = 3) and 5 rats were randomly selected from Sham group and SAH group respectively (n = 5). The fluorescence dyes and EB dye were applied as tracers of ISF for exploring its drainage characteristic under the TPF microscope. The fluorescence tracers ALEXA-594 hydrazide (A594, 759 D) and FITC-dextran 2000 (FITC-d2000, 2000 kD) were constituted in artificial CSF at a final concentration of 0.05% (all reagents were from Invitrogen, Oregon, USA), and Cascade Blue (CB, 10 kD) was injected into tail vein to show the microvessels. Briefly, the rat was placed in the stereotaxic frame, and the atlantooccipital membrane was exposed, a Hamilton syringe needle (50 μL, Hamilton, Reno, Nevada) was inserted 1 mm depth into the cisterna magna. The 25 μL EB (2%, Sigma-Aldrich, Darmstadt, Germany) or fluorescence tracer commixture of A594 and FITC-d2000 (50 μL) were injected into cisterna magna (5 μL / min) of the animals. At 0.5 h after injection, the rats were anesthetized and intracardially perfused with normal saline (0.9%, 4 °C) and paraformaldehyde (4%, 4 °C). Brains were postfixed in 4% polyformaldehyde for 8–12 h. The fixed tissue blocks were then cryoprotected in 30% sucrose. A freezing microtome (Leica, 1900, Wetzlar, Germany) was used to obtain coronal brain sections (20 μm), and the sections were stained by Hematoxylin–Eosin (HE), immunofluorescence staining or TUNEL staining.

### HE staining and evaluation of PVS area

Three slices were randomly selected from Sham group and SAH group of ISF tracking respectively, and then the sections were stained with hematoxylin for 5 min, stained with eosin for 3 min. The sections were mounted using the synthetic resin (Entellan; Merck, Darmstadt, Germany). The ratio of PVS area to the whole microvessel area (diameters of approx 20 μm) was calculated and used for assessing the PVS injury after SAH. The specific method and calculation formula are shown in the Fig. 4B.

### Immunofluorescence staining

Three slices were randomly selected from Sham group and SAH group of ISF tracking respectively, and the immunofluorescence staining was performed as reported by others [7]. Briefly, brain sections including frontal cortex were incubated with anti-GFAP (1:300, ab7260, Abcam, Cambridge, UK), anti-α-SMA (1:200, A5228, Sigma-Aldrich, Darmstadt, Germany), anti-MMP9 (1:200, ab76003, Abcam, Cambridge, UK), anti-COL4A3 (1:200, ABIN678128, Antibodies-online, Aachen, Germany), and corresponding secondary antibodies (Alexa Fluor 488 goat anti-mouse, 1:500, #4408, Cell Signaling Technology, Massachusetts, USA; Alexa Fluor 488 goat anti-rabbit, 1:500, #4412, Cell Signaling Technology, Massachusetts, USA). The images of the staining were captured using a Laser Scanning Confocal Microscope (LSCM) SP8 (Leica, Heerbrugg, Germany).

### TUNEL staining

Three slices were randomly selected from Sham group and SAH group of ISF tracking respectively, and then TUNEL staining method was applied to detect the apoptosis of endothelial cells. The brain tissue sections containing cortex and hippocampus were stained using a TUNEL Kit (Roche, New York, USA), and TUNEL-positive cells were revealed by fluorescein-dUTP with dNTP and POD with DAB.

### Western blot

Five rats were randomly selected from Sham group and SAH group respectively (n = 5), and animals in deep anesthesia were perfused through the left ventricles with 200 mL ice-cold 0.1 mol/L PBS. After the brain tissue was obtained, it was divided into two parts, and one was kept at − 80 °C until analysis, the other immediately underwent MMP9 zymography. A BCA Protein Assay Kit (P1511, Applygen, Beijing, China) was used to determine the protein concentrations of the samples. 10 – 20 g of total protein was separated using 8 – 12% sodium dodecyl sulfate-polyacrylamide gel. The proteins present in the gel were shifted to nitrocellulose membranes via liquid electroblotting. The membranes were subsequently rinsed in Tris-buffered saline with 0.05% Tween 20 (TBST; pH 7.4) then soaked in blocking buffer (5% bovine serum albumin [BSA] in TBST) for 1 h at room temperature. Anti- MMP9 (1:1000, ab76003, Abcam, Cambridge, UK), anti-COL4A3 (1:2000, ABIN678128, Antibodies-online, Aachen, Germany) and anti-β-Actin (1:1000, #3700, Cell Signaling Technology, Massachusetts, USA) primary antibodies were used for incubation overnight, respectively, in TBST containing 5% BSA on a shaker at 4 °C. The next day, the membranes were rinsed, soaked with corresponding goat anti-rabbit (1:20000, A0545, Sigma-Aldrich, Darmstadt, Germany) or goat anti-mouse (1:20000, A0168, Sigma-Aldrich, Darmstadt, Germany) IgG secondary antibody dissolved in TBST containing 5% BSA on a shaker at room temperature for 2 h. Finally, the membranes were infiltrated with Super Signal Enhanced Chemiluminescence Substrate (#1705060, Bio-Rad, California, USA). Chemiluminescent signals were detected using the gray value of bands and quantified with Image Pro Plus 7 software. After getting the absolute gray value of each band, the relative gray value was obtained by the absolute gray value of the target protein/the absolute gray value of the internal reference protein [37].

### MMP9 zymography

The MMP9 gelatinase activity was determined by zymography using a gelatin zymography assay kit (P1700, Applygen, Beijing, China) as described previously [30]. Briefly, the brains obtained above were homogenized in Lysis buffer on ice. Total protein concentrations were determined using the Bradford assay (Bio-Rad, California, USA). Equal amounts (50 μg) of total protein extract were prepared and MMP9 activity was measured by SDS-PAGE under non-reducing conditions. The gel contained 1% gelatin and 30% acrylamide. Electrophoresis was carried out at 4 °C. After washing with buffer A (containing 2% Triton X-100) from the kit, the gel was incubated at 37 °C with buffer B (containing the necessary metal ion: 5 mmol/CaCl_2_, 1 μmol/L ZnCl_2_). MMP activity was visualized by staining with Coommasie Blue R-250 (P1501, Applygen, Beijing, China). Signals were detected using the gray value of bands and quantified with Image Pro Plus 7 software. After obtaining the absolute gray value of each band, the relative gray value of SAH group was obtained by the absolute gray value of the SAH group / the absolute gray value of the Sham group.

### TEM scanning

Five rats were randomly selected from Sham group and SAH group respectively (n = 5). At 24 h after SAH, 2 μL colloidal gold solution was injected into frontal cortex of the animals. At 3 h after injection, the sample was fixed in 3% glutaraldehyde for 24 h and stained with 2% osmium tetroxide for 1 h, and dehydrated in the graded alcohols. The sample was then embedded in plastic (Araldite based). The thin sections were made and stained with uranyl acetate/lead citrate. The sections were placed on the copper grids and imaged by using a JEOL J EM-100S electron microscope.

### MRI scanning

Five rats were randomly selected from Sham group and SAH group respectively (n = 5) for these experiments. The MRI tracer Gd-DTPA is impermeable to the neuronal membrane and evenly distributed within ISF, and had been demonstrated as a good indicator for ISF drainage [32]. In this study, Gd-DTPA was diluted to 10 mmol/L in normal saline solution and injected into the frontal cortex of rats. The rat was anesthetized as above, and the body temperature was maintained at 37 ± 0.5 °C. An incision was performed to expose the bregma of the skull. A small hole was made at the site of frontal cortex area. A 2 μL Gd-DTPA solution was injected at rate of 0.2 μL / min. The rats were quickly placed in the scanner with a prone position for the MRI scaning. A 3.0 T MRI system (Magnetom Trio, Siemens Medical Solutions, Germany) with a small animal-specific coil was applied to obtain the brain images using T1-weighted magnetization-prepared rapid-acquisition with a gradient echo sequence. The acquisition parameters were as follows: echo time, 3.7 ms; repetition time, 1,500 ms; flip angle, 9°; inversion time, 900 ms; field of view, 267 mm; voxel, 0.5 mm^3^; matrix, 512 × 512; number of averages, 2; and phase-encoding steps, 96. The acquisition time for each rat was 290 s. MRI was also conducted before Gd-DTPA injection to exclude the congenital defects in brain. The images were recorded at 15, 30, 45, 70, 110, 150 min following Gd-DTPA injection.

The image post-processing was performed according to the previous report [19]. The post-injection images were compared with the baseline images following the grayscale calibration, mutual information-based image registration and histogram equalization. The images were also subjected to rigid transformation, similarity measurements, high-order interpolation and adaptive stochastic gradient descent optimization. In addition, these images were subtracted from the pre-scanned images. The coronal MRI images with 1 mm slice thicknesses were generated by the software. The ISF diffusion and extracellular space (ECS) structural parameters were calculated based on the distribution of Gd-DTPA concentration using the Nano Imaging Detect Analyze system software (Version 2.1, MRI lab, Beijing, China). The change of ISF clearance parameter k', half life time (T_1/2_) and ECS microstructure parameters D* (effective diffusion coefficient) was calculated and analyzed between the Sham and SAH groups.

### Statistical analysis

The data was expressed as mean ± standard deviation (SD). Statistical analyses were performed using SPSS 19.0 software (SPSS Inc., Illinois, USA). One-way analysis (ANOVA) and Tukey's multiple comparison test were applied for data comparison. A P-value less than 0.05 was statistically considered significant.

## Results

### Distribution of tracers along the IPAD pathway

**Fig. 2:**
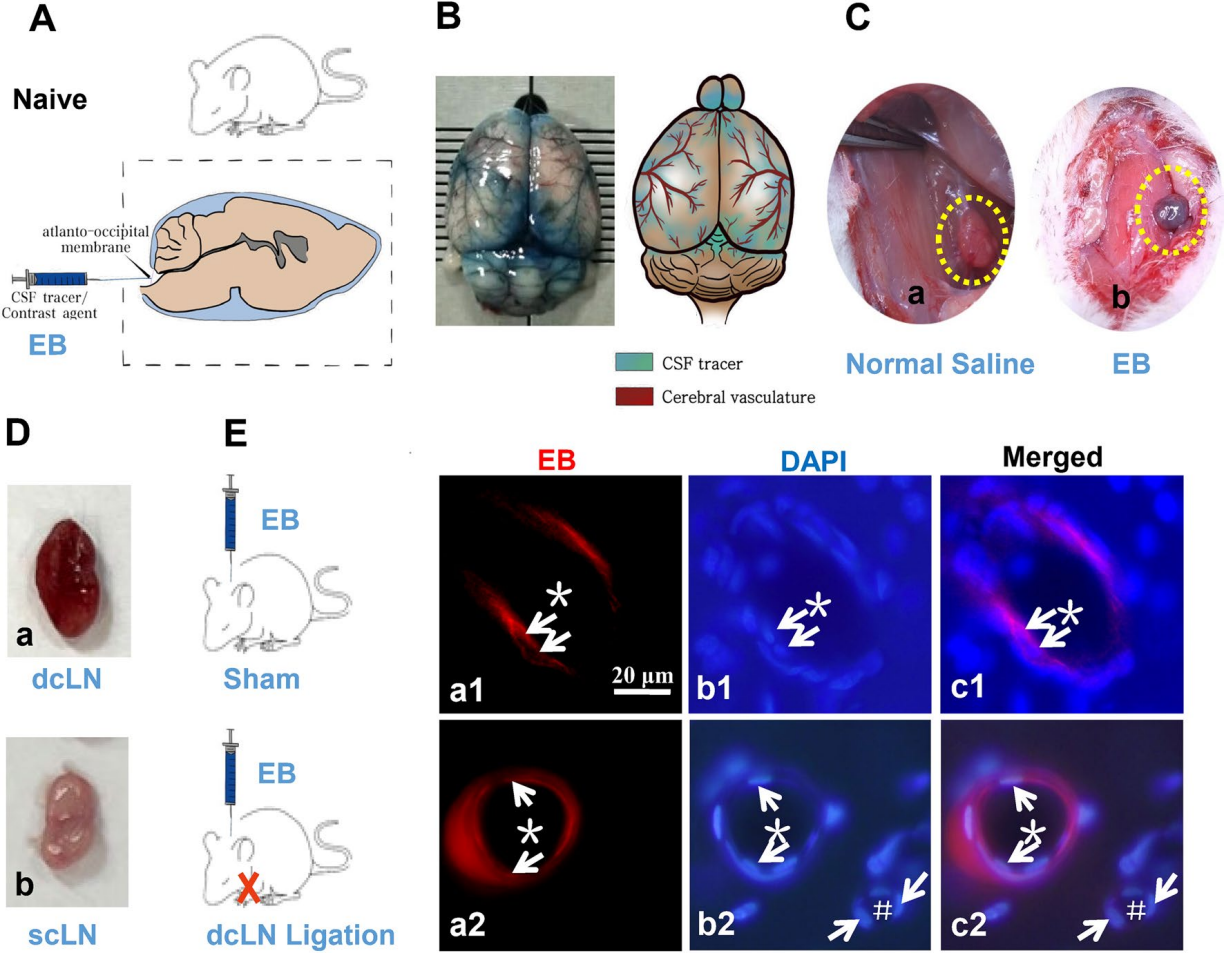
The exploration of IPAD pathway using EB. At 0.5 h after EB was injected into cisterna magna of Naive group and dcLN ligation group rats, n = 3 per group (**A**), and EB was mainly distributed along with PVS around middle cerebral artery on the brain convex surface and the circle of Willis on the basal surface of the brain (**B**). EB in the brain parenchyma was detected within dcLNs and not in the superficial scLNs (**C**, **D**). EB in the ECS was observed along the basement membrane of tunica media in the capillaries (**E**.a1–c1). After ligation of dcLNs, EB was found accumulated in the basement membrane of the arterioles possibly due to the blockage of IPAD pathway (E.a2-c2). In figures C, the circle with yellow dotted lines represents the dcLN. In figures E, the arrow represents an endothelial cell; “*” represents arteriolar lumen;“#” indicates venular lumen. Scale bar = 20 μm. Abbreviations: dcLN, deep cervical lymph node; EB, Evans Blue; ECS, extracellular space; IPAD, intramural periarterial drainage; PVS, perivascular space; scLN, superficial cervical lymph nodes

Firstly, we injected EB into cisterna magna (Fig. 2A). At 0.5 h after injection, EB was mainly distributed along the PVS of the middle cerebral artery on the brain convex surface and Willis cycle on the base of the brain (Fig. 2B). EB was also observed into the dcLNs, not in the superficial cervical lymph nodes (scLNs) (Fig. 2C, D). Noticeably, EB was observed within the ECS of the brain mainly in the basement membranes of the capillaries (Fig. 2E.a1–c1). Furthermore, after ligation of dcLNs, EB was accumulated in the basement membranes of arterioles (Fig. 2E.a2–c2).

### Characteristic of IPAD drainage

**Fig. 3:**
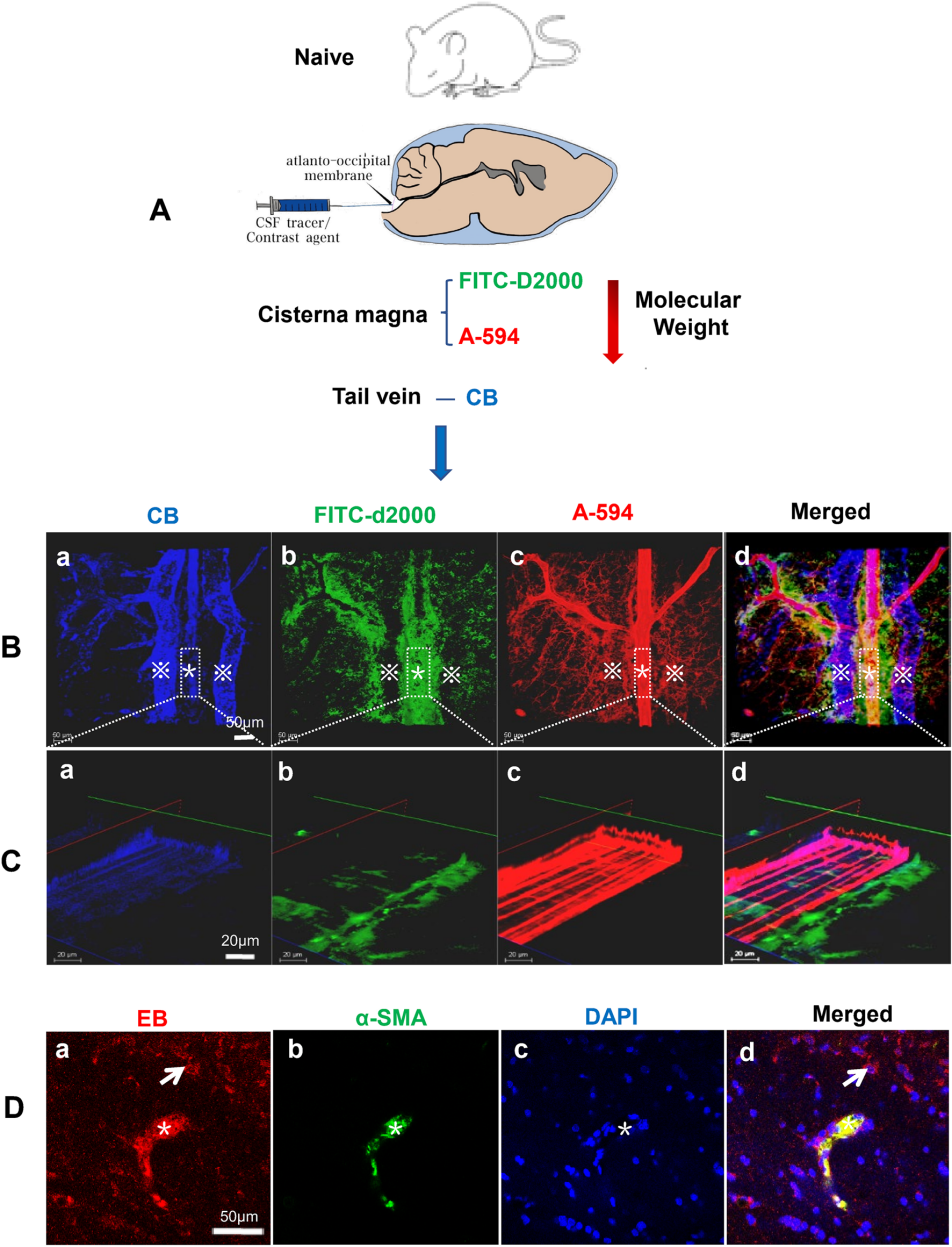
The different drainage patterns of the fluorenscence dyes. Two fluorenscence dyes, FITC-D2000 (molecular weight 2000 kD) and A-594 (molecular weight 759 D) were injected into cisterna magna of Naive group rats, and the CB dye was injected into tail veins to show the microvessels, n = 3 (**A**). At 0.5 h after injection, both FITC-D2000 and A-594 flowed into the brain parenchyma along the PVS. The higher molecular weight, FITC-D2000 was predominantly distributed in PVS around the arterioles, and was engulfed by surrounding astrocytes and microglia. Only a small amount of FITC-D2000 was observed along the basement membrane of the capillaries (**B**.b, **C**.b). The low molecular weight A-594 dyes were mainly cleared along the basement membranes, and some A-594 dyes were also engulfed by the activated glia cells (B.c, C.c). (B.b-d, C.b-d). α-SMA labeling of microvessels containing EB confirmed that the microvessels in this study were a arterioles (**D**.a-d). **C**.a-d are the fluorescence intensity statistical images of the microvessels showed in **B**.a-d. In figures **B**.a-d, “※” represents the venule; “*” indicates the arteriole. In figures **D**.a-d, “*” indicates the arteriole; “arrow” represents the glial cell that engulfed EB. In figures B.a-d, scale bar = 50 μm; in figures **C**.a-d, scale bar = 20 μm; in figures **D**.a-d, scale bar = 50 μm. Abbreviations: A-594, ALEXA-594 hydrazide; α-SMA, Alpha-smooth muscle actin 2; CB, Cascade Blue; EB, Evans Blue; FITC-D2000, FITC-dextran 2000; PVS, perivascular space

To investigate the outflow characteristic of the solutes with different molecular weight through IPAD pathway, two fluorenscence dyes, FITC-D2000 (2000 kD, simulating macro-molecule methemoglobin in blood lysates) and A-594 (759 D, simulating micro-molecule heme in blood lysates) were injected into cisterna magna to observe their clearance from the frontal cortex at 0.5 h after injection (Fig. 3A).

The results showed that, under the physiological condition, both FITC-D2000 and A-594 flowed into brain along he PVS. FITC-D2000 was predominantly distributed in PVS compartment, and was partially engulfed by the surrounding astrocytes and microglia. Only a small amount of FITC-D2000 was observed along the basement membranes of the capillaries (Fig. 3B.b, C.b). The smaller molecular weight A-594 dye was mainly observed within the basement membrane of the capillaries, and some A-594 dye was observed within glial cells (Fig. 3B.c, C.c). Labelling smooth muscle cells of arterioles showed that EB was mainly associated with arterioles rather than venules (Fig. 3D.a–d).

### Impairment of IPAD following SAH

**Fig. 4:**
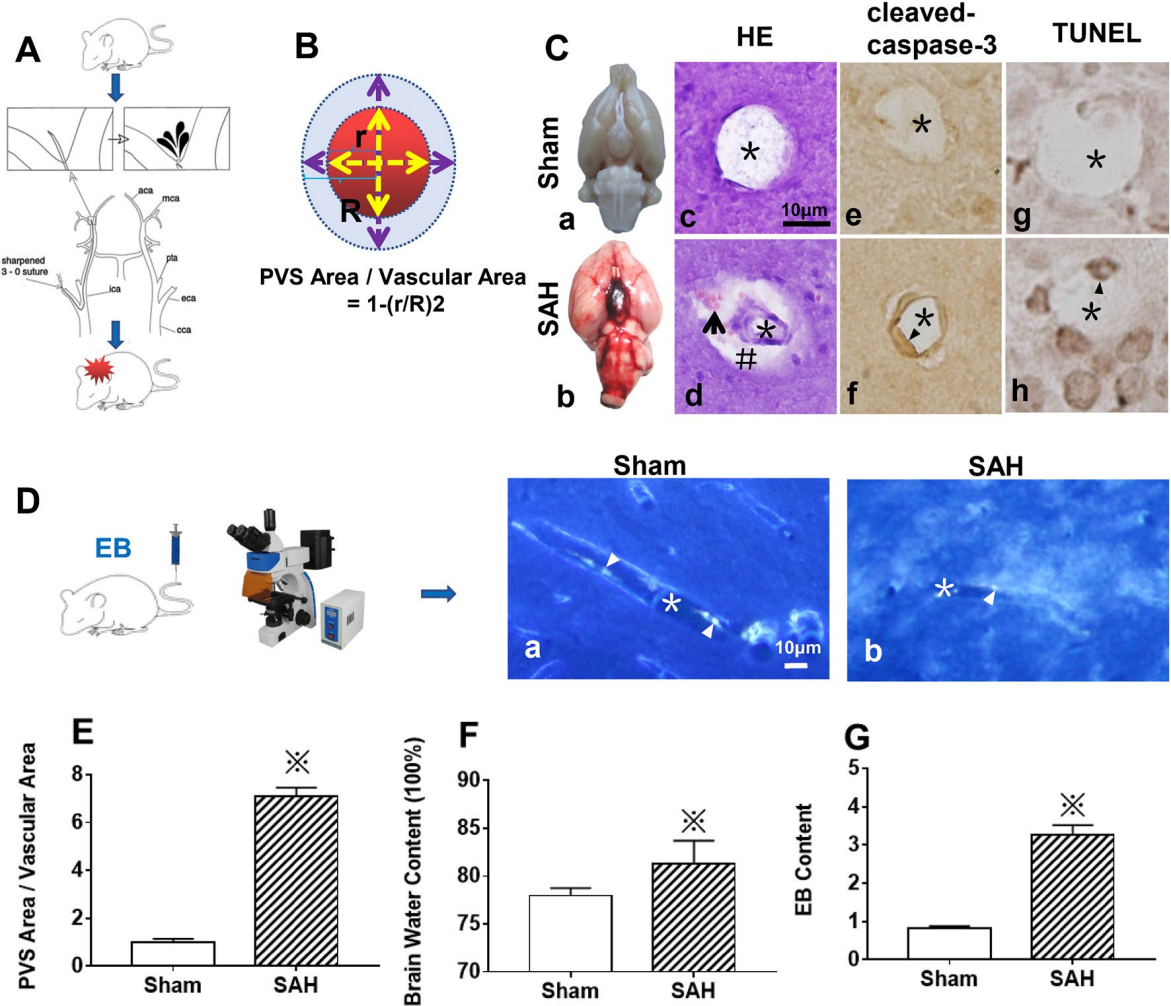
The impairment of PVS and brain edema after SAH. The protocol for establishing SAH model is shown in (**A**). The method for calculating the ratio of PVS to the microvessel was showed in (**B**), “r” indicating the radius of the microvessel after SAH, “R” indicating the radius of the microvessel under physiological state. The PVS% = PVS Area / Vascular Area = π (R^2^ − r^2^) / πR^2^ = 1 − (r/R)^2^. The PVS% parameter was significantly increased following SAH, which implied that there was marked blockage in the ISF flow (**E**). After SAH, there were blood clots in the subarachnoid space (**C**.a,b). The haematoxyin-eosin staining showed that, compared with those of Sham group, the diameters of the microvessels were markedly reduced and the PVS around the microvessels was significantly expanded in SAH group. After SAH, blood cells entered into the PVS from CSF (**C**.c,d), additionally, the expression level of apoptotic executor protein cleaved-caspase-3 (**C**.e,f) and the number of apoptotic endothelial cells (**C**.g,h) were significantly increased, which led to capillary injury and BBB disruption, as indicated by EB leakage from BBB (D, G) and brain edema formation(78.3% ± 0.4% vs. 81.2% ± 0.8%, **F**). “※” in (**E**), (**F**) and (**G**) indicated *P* < *0.05* compared with that of Sham group, n = 5 per group. “*” indicating the microvessel lumen; “arrow” pointing to the blood cell in PVS; “#” showing the PVS; “▲” indicating the endothelial cell. Scale bar = 10 μm. Abbreviations: BBB: blood brain-barrier; CSF: cerebrospinal fluid; ECS: extracellular space; IPAD: intramural periarterial drainage; ISF: interstitial fluid; PVS,:perivascular space; SAH: subarachnoid hemorrhage

After SAH, prominent blood clots were observed in the subarachnoid space, especially in the basal cistern (Fig. 4A, C.a,b). The HE staining results showed that, compared with those of Sham group, the diameters of the capillaries were markedly reduced accompanied by PVS expansion (Fig. 4B, C.c,d, E). In addition, numerous blood cells were observed in the PVS associated with SAH (Fig. 4C.c,d). After SAH, the expression level of apoptotic executor protein cleaved-caspase-3 (Fig. 4C.e,f) and the number of apoptotic endothelial cells (Fig. 4C.g,h) were significantly increased.

EB is an azo dye with high affinity for plasma albumin. Under physiological conditions, the combination of EB and albumin in blood is strictly confined into the microvasculature due to the blood–brain barrier (BBB). In the experimental group, some EB was engulfed by the endothelium and EB was also observed in the brain parenchyma (Fig. 4D.a). After SAH, EB was extravasated through a disrupted BBB into the brain parenchyma and widely distributed in the perivascular parenchyma, and the EB content was also significantly increased compared with that of Sham group (Fig. 4D.b, G).

Furthermore, since PVS appeared to contain CSF after SAH, we measured the whole brain water content. The results indicated that the brain water content in SAH group was significantly increased compared with that of Sham group (81.2% ± 0.8% vs. 78.3% ± 0.4%, Fig. 4F).

**Fig. 5:**
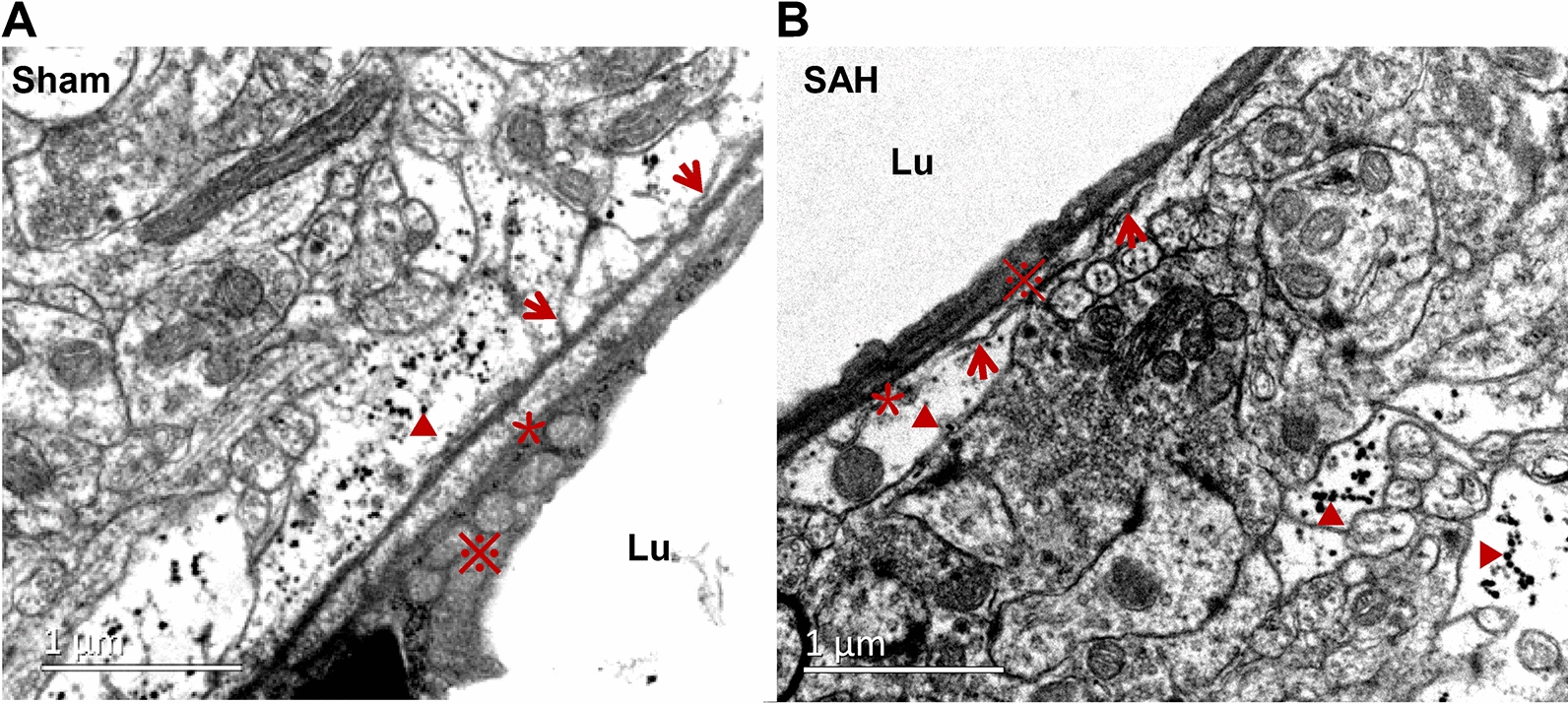
The ultrastructural changes of IPAD following SAH. In the Sham group, at 3 h after the nanoparticles injection into the frontal cortex, the junctions of ECS with CVBM at the site of the astrocyte membrane were clearly visible and were almost perpendicular to the CVBM (arrows). The nanoparticles were distributed adjacent to the CVBM. After SAH, the number of the junctions was markedly decreased, and most of them were parallel with CVBM. The clustered nanoparticles were mainly distributed distant to the capillary, with only a few punctate nanoparticles scattered around the CVBM. n = 5 per group. “▲” indicates the nanoparticle; “*” indicates the CVBM; “※” shows the endothelial cells, “arrow” indicates the ECS. Scale bar = 1 μm. Abbreviations: CVBM: capillary vessel basement membrane; ECS: extracellular space; IPAD: intramural periarterial drainage; Lu: microvessel lumen; SAH: subarachnoid hemorrhage

At 24 h after SAH, the 10 nm nanoparticles were injected into the frontal cortex. Three hours later, the distribution pattern of nanoparticles was revealed by using TEM. In the Sham group, the junctions of ECS with the capillary basement membrane (CBM) at the site of the astrocyte membrane were clearly showed, which were almost perpendicular to the capillary basement membrane. The clustered nanoparticles were distributed abutting the capillary basement membrane. After SAH, the number of such junctions was markedly decreased, and most of them were parallel with capillary basement membrane. The clustered nanoparticles were mainly distributed distant to the capillary, with only a few punctate nanoparticles scattered around the basement membrane (Fig. 5A, B).

**Fig. 6:**
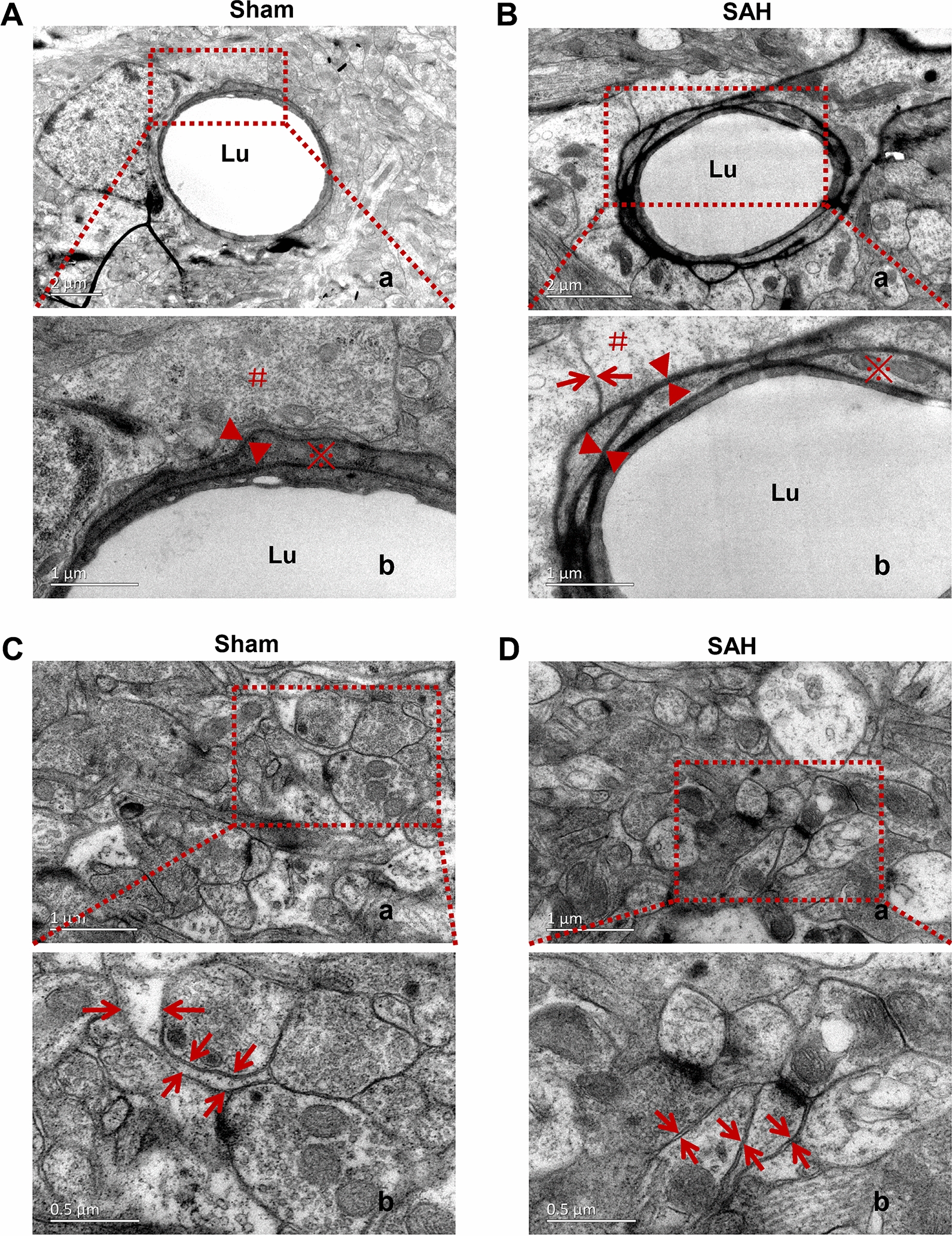
Narrowing of the ECS following IPAD injury after SAH. Compared with that of Sham group (**A**), the width of the IPAD pathway was significantly increased after SAH (**B**), suggesting IPAD impairment. The ECS around the capillaries in the frontal cortex were markedly narrowed after SAH due to the defects in the basement membranes and astrocyte swelling (**A**, **B**, **C**, **D**). n = 5 per group. **A**.b, **B**.b, **C**.b and **D**.b are enlarged images of the framed areas in A.a, B.a, C.a and D.a, respectively. “Arrow” indicates ECS; “▲” indicates the IPAD; “#” indicates the endfoot of astrocyte; “※” shows the endothelial cells. A.a, B.a: scale bar = 2 μm; **A**.b, **B**.b, **C**.a, **D**.a: scale bar = 1 μm; **C**.b, **D**.b: scale bar = 0.5 μm. Abbreviations: ECS: extracellular space; IPAD: intramural periarterial drainage; SAH: subarachnoid hemorrhage; Lu: microvessel lumen

Furthermore, compared with that of Sham group, the width of the capillary basement membranes (effectively IPAD pathways) after SAH was significantly increased (Fig. 6A, B). In addition, the ECS in frontal cortex around the capillary was markedly narrowed (Fig. 6C, D).

**Fig. 7:**
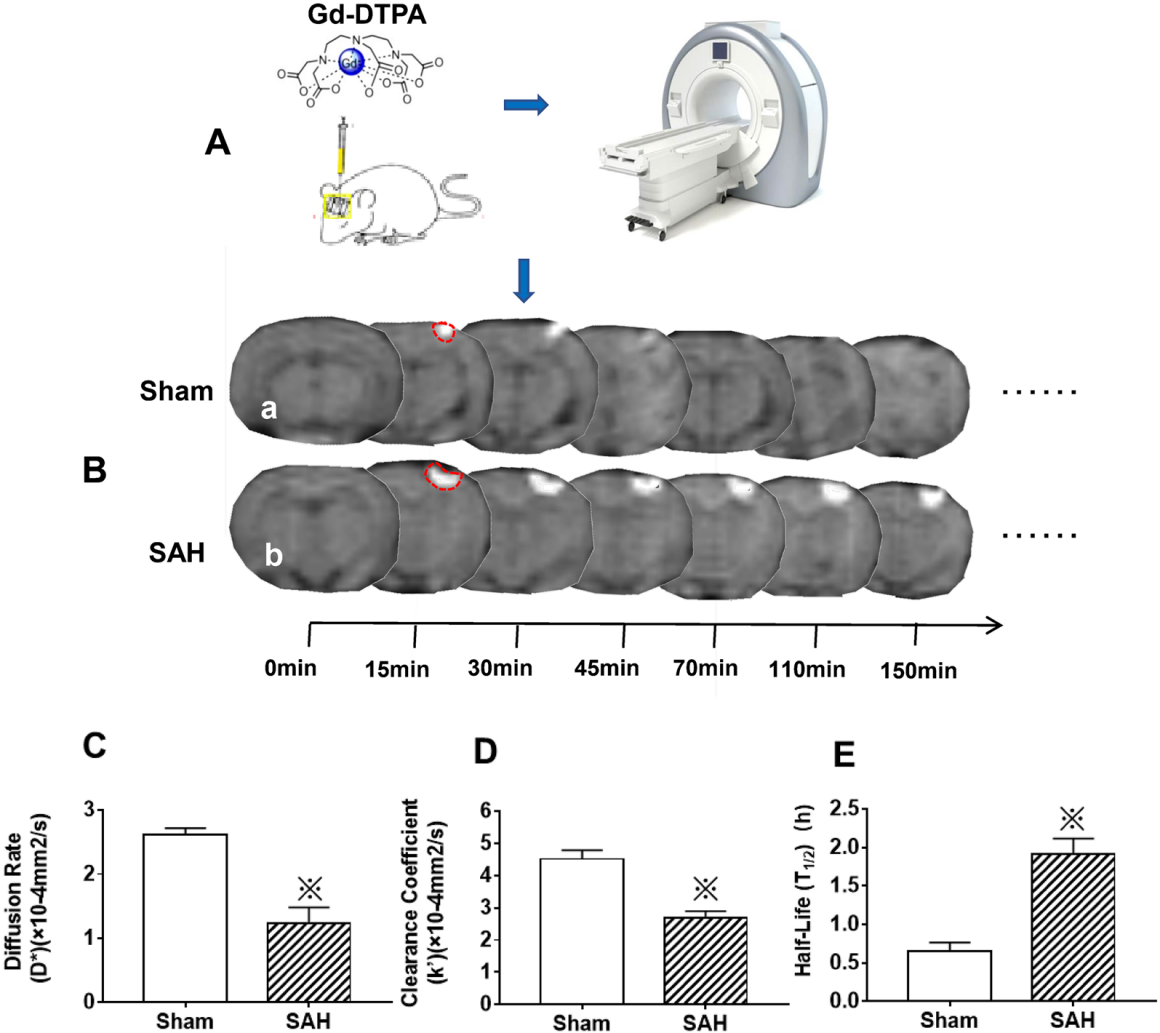
The ISF flow injury detected by MRI tracking technique after SAH. Following Gd-DTPA injection (**A**), the diffusion rate (D*), clearance rate (k') and the half time of Gd-DTPA were measured at 0, 15, 30, 45, 70, 110, 150 min. The results indicated that, the signal of Gd-DTPA in cortex was gradually weakened, and in the Sham group, the Gd-DTPA was completely cleared from brain at 110 min following injection; however, the signal intensity of Gd-DTPA was still strong at 150 min in the SAH group (**B**). After SAH, the Gd-DTPA diffusion rate (D*) and clearance rate (k') were significantly decreased compared with those of Sham group: consequently, the half time(T_1/2_) of Gd-DTPA signal intensity was markedly extended (**C**, **D**, **E**). These results showed that there was impairment of ECS spatial conformation and ISF drainage in the ECS after SAH. “※” in C, D and E indicate *P* < 0.05 compared with that of Sham group, n = 5 per group. Abbreviations: ECS: extracellular space; Gd-DTPA: gadolinium-diethylenetriaminepentaacetic acid; IPAD: intramural periarterial drainage; ISF: interstitial fluid; MRI: magnetic resonance imaging; SAH: subarachnoid hemorrhage

To further validate these results and understand their significance, the diffusion rate (D*), clearance rate (k') and the half time of Gd-DTPA were measured following Gd-DTPA injection (Fig. 7A). The results indicated that the signal of Gd-DTPA in cortex was gradually weakened, and in Sham group, the Gd-DTPA was completely cleared from brain at 110 min following injection; however, the signal intensity of Gd-DTPA was still strong at 150 min (Fig. 7B). In contrast, after SAH, the Gd-DTPA diffusion rate (D*) and clearance rate (k') were significantly decreased compared with those of Sham group, with the half time (T_1/2_) of Gd-DTPA signal intensity markedly extended (Fig. 7C, D, E).

**Fig. 8:**
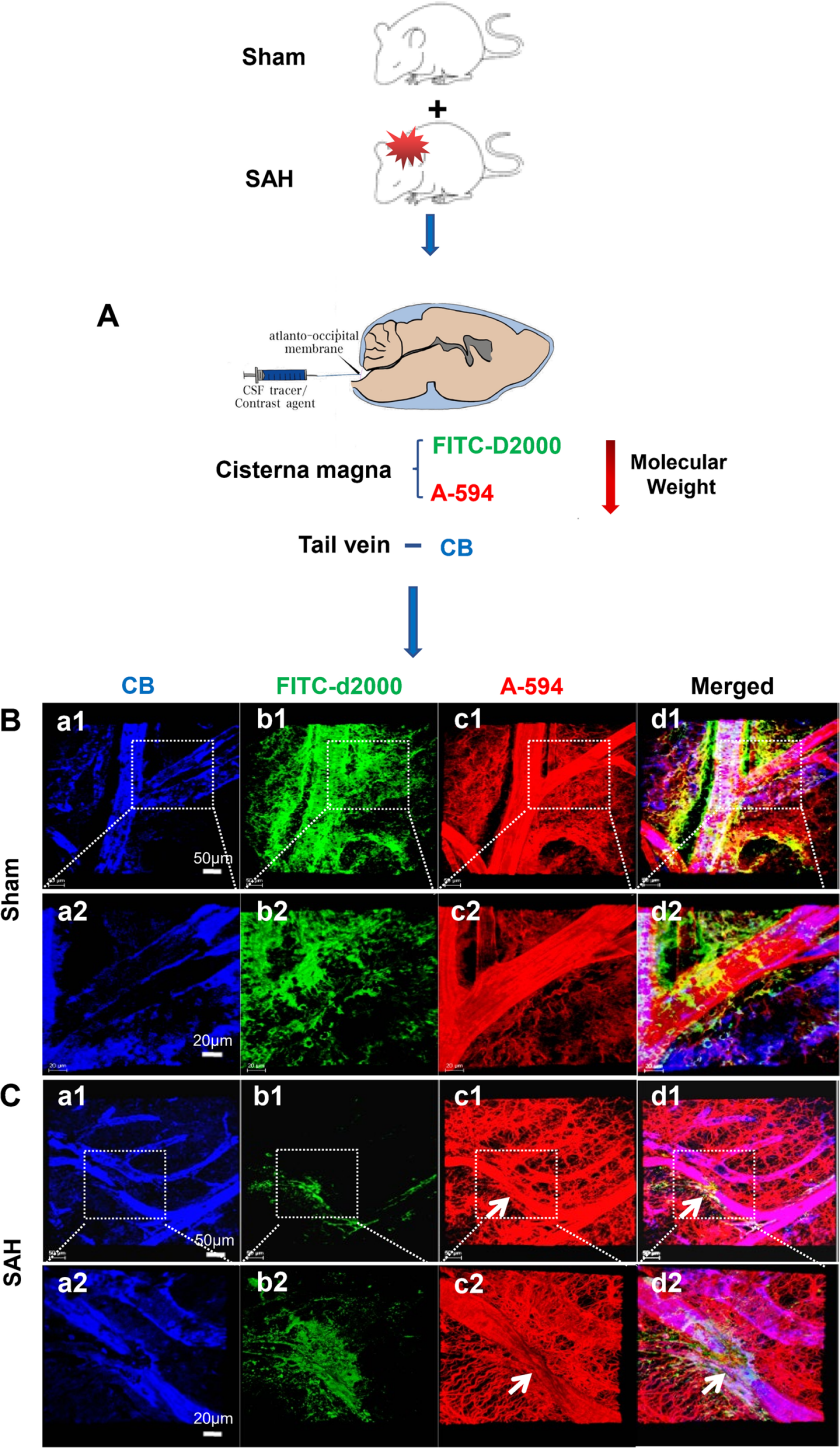
The impairment of IPAD drainage of the fluorenscence dyes. At 24 h after SAH, the FITC-D2000 and A-594 dyes were injected into cisterna magna, and the CB dye was injected into tail veins to show the microvessels in the cortex, n = 5 per group (**A**). At 1 h following injection, in the frontal cortex, both FITC-D2000 and A-594 flowed into the ECS and their clearance rates through IPAD were both markedly decreased compared with those of Sham group (**B**.b1, b2, c1, c2, **C**. b1, b2, c1, c2). The astrocytes surrounding the arterioles had thickened and distorted end-feet (**C**). **B**.a2-d2 are the magnified images of the framed areas in **B**.a1–d1; **C**.a2–d2 are the magnified images of the framed areas in **C**.a1-d1. “Arrow” in **C**.c1–d1, c2–d2 shows vasospasm in the arterioles after SAH. **B**.a1–d1, **C**.a1–d1: scale bar = 50 μm; **B**.a2–d2, **C**.a2–d2: scale bar = 20 μm. Abbreviations: A-594: ALEXA-594 hydrazide; CB, Cascade Blue; EB: Evans Blue; ECS: extracellular space; FITC-D2000: FITC-dextran 2000; IPAD: intramural periarterial drainage; SAH: subarachnoid hemorrhage

To investigate the changes of clearance rates after SAH, at 24 h after SAH, the FITC-D2000 and A-594 dyes were injected into cisterna magna, and the CB dye was injected into tail veins to mark the microvessels (Fig. 8A). Following SAH, the clearance rates of FITC-D2000 and A-594 from brain parenchyma through IPAD were markedly decreased compared with that of Sham group (Fig. 8B.b1–d1, b2–d2, C.b1–d1, b2–d2). These morphological and functional injury of the IPAD pathway were associated with PVS expansion as shown in Fig. 4B, C.c,d, E, and Fig. 6A, B. The astrocytes surrounding the capillaries had thickened and distorted endfeet (Fig. 8B, C).

### Potential mechanism of PVS injury after SAH

**Fig. 9:**
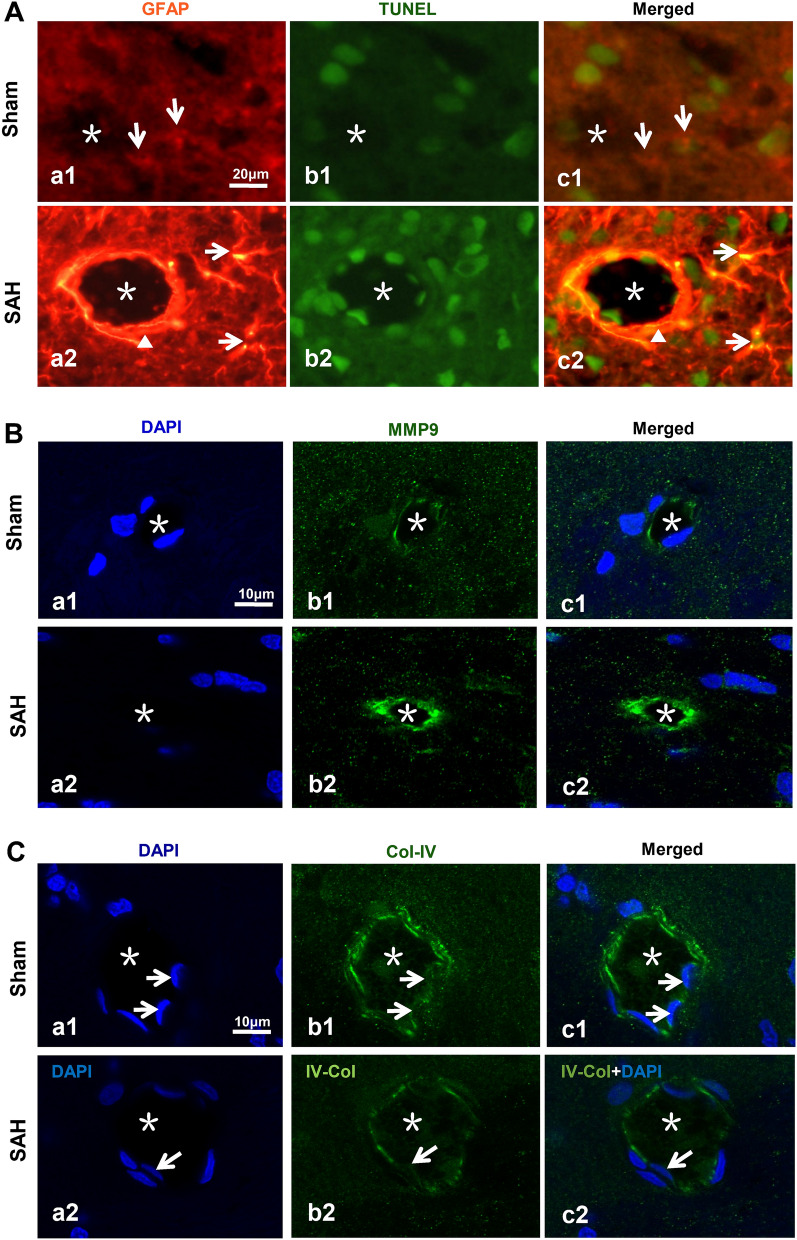
The molecules involved in IPAD impairment after SAH. In the Sham group, the astrocytes were evenly distributed around the microvessels, and there were rare apoptotic astrocytes in cortex (**A**.a1–c1). After SAH, the expression level of GFAP was markedly enhanced, and there were numerous apoptotic astrocytes around the microvessels (**A**.a2–c2). The number of astrocyte processes was increased after SAH (**A**.a1, a2, c1, c2). Immunofluorescence staining showed that after SAH, the endothelial MMP9 of microvessels increased significantly (**B**.a1–c1, a2–c2), while Col-IV decreased significantly (**C**.a1–c1, a2–c2). The nuclei were labeled with DAPI (B.a1, a2, **C**.a1, a2). n = 5 per group. “Arrow” in A shows the astrocyte; “*” indicates the microvessel lumen; “▲” indicates the microvessels; “Arrow” in **C** showed the nucleus. A: scale bar = 20 μm; B, C: scale bar = 10 μm. Abbreviaitons: DAP: 4',6- diamidino-2- phenylindole; Col-IV: Collagen Type IV; GFAP: glial fibrillary acidic protein; IPAD: intramural periarterial drainage; MMP9: Matrix metalloproteinase 9; SAH: subarachnoid hemorrhage

We investigated the potential mechanism for the failure of IPAD after SAH. Under physiological conditions, the astrocytes were evenly located around the microvessels,and rare apoptotic astrocytes were observed (Fig. 9A.a1–c1). After SAH, the expression level of glial fibrillary acidic protein (GFAP) was markedly enhanced. There were numerous apoptotic astrocytes following SAH (Fig. 9A.a2–c2). After SAH, the immunofluorescence expression level of MMP9 in endothelial cells of the microvessels was significantly increased (Fig. 9B) while the Col-IV expression in the basement membrane was accordingly decreased (Fig. 9C).

**Fig. 10:**
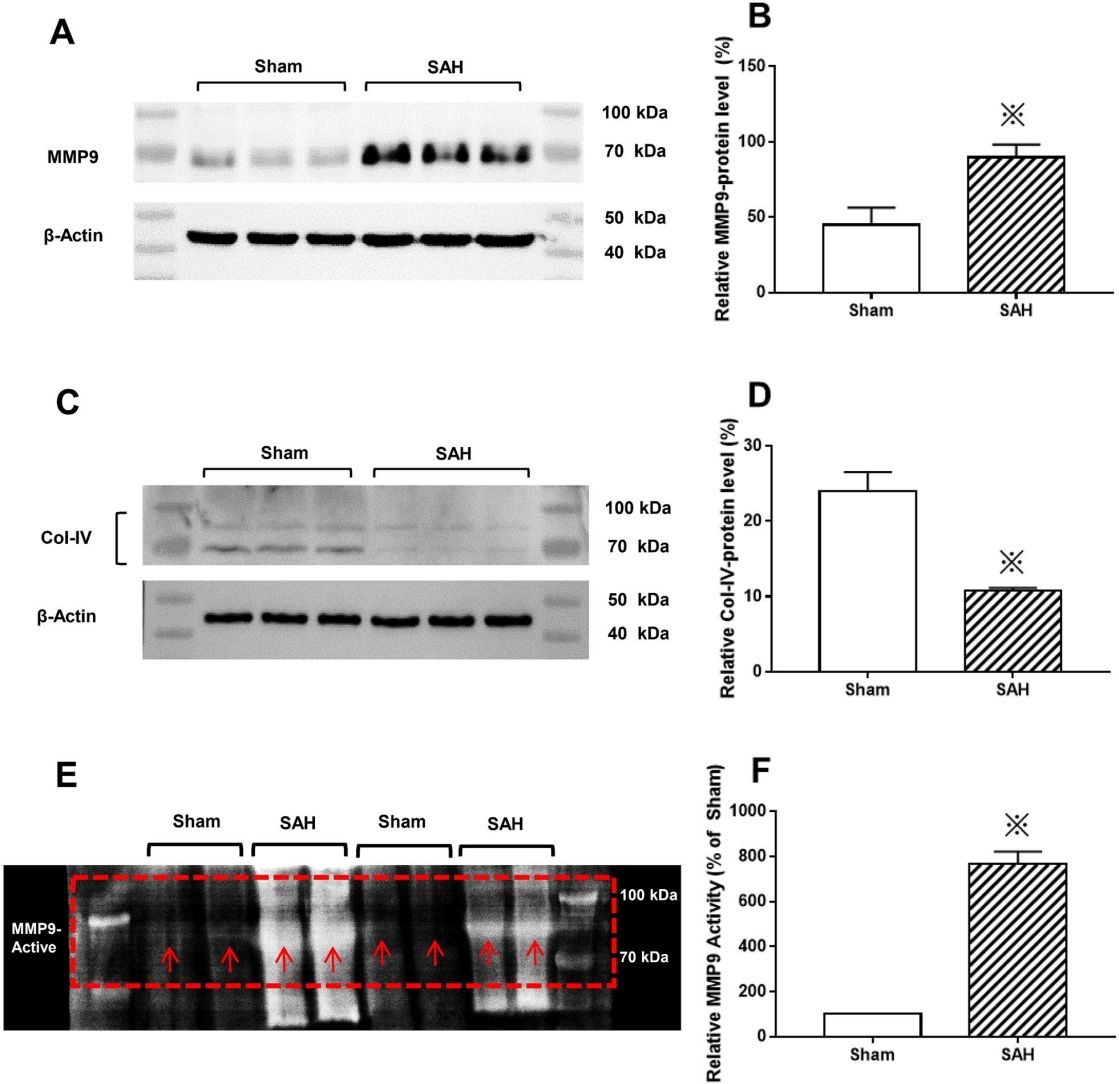
The expression of activated MMP9 and Col-IV after SAH. The MMP9 protein expression level was markedly increased in the SAH group compared to those of Sham group (**A**, **B**). The level of Col-IV protein was significantly decreased in the SAH group compared to that of Sham group (**C**, **D**). Compared with that of Sham group, the enzymatic activity of MMP9 in SAH group was significantly enhanced (**E**, **F**). “※” in B, D and F indicating *P* < 0.05 compared with that of Sham group, n = 5 per group. “Square box” indicating representative MMP9 zymography; “Arrow” in E showing the activated MMP9. Abbreviations: GFAP: glial fibrillary acidic protein; IPAD: intramural periarterial drainage; Col-IV: Collagen Type IV; MMP9: matrix metalloproteinase 9; SAH: subarachnoid hemorrhage

A similar pattern for the expression of MMP9 and Col-IV was detected also by western blot (Fig. 10A, C). The MMP9 protein expression level was markedly increased in SAH group compared to those of Sham group (Fig. 10A, B). The level of Col-IV protein was significantly decreased in SAH group compared to that of Sham group (Fig. 10C, D). Zymography was performed at 24 h after SAH. Representative MMP9 zymography is shown in Fig. 10E. Compared with that of Sham group, the enzymatic activity of MMP9 in SAH group was significantly enhanced (Fig. 10F).

## Discussion

**Fig. 11:**
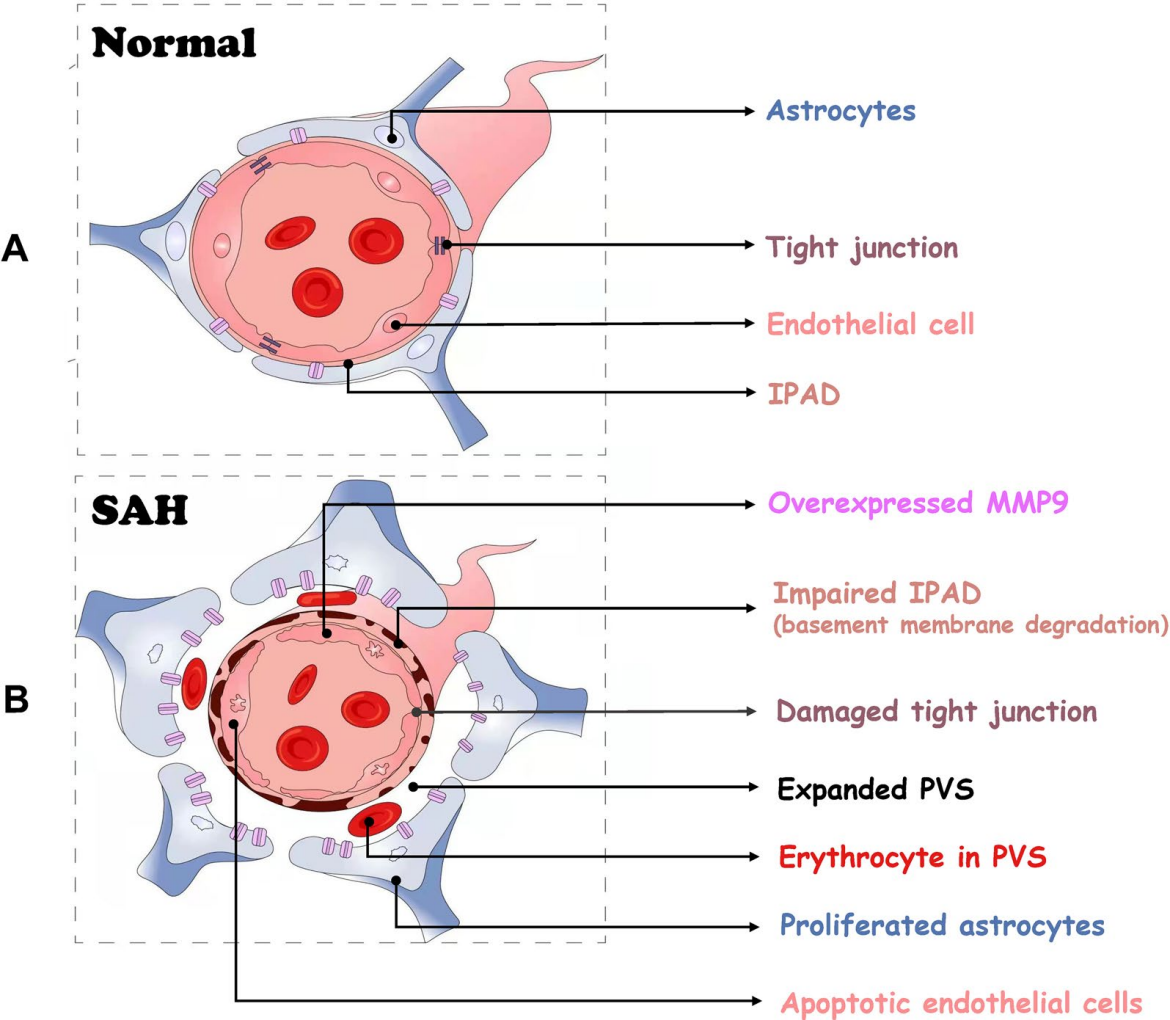
The potential mechanism of IPAD impairment after SAH. Under physiological state (**A**), the ISF and solutes leave the brain, at least partly, along the basement membranes in the walls of capillaries and arteries (IPAD pathway). After SAH (**B**), there is a marked impairment in IPAD system, such as expanded PVS, disruption of tight junctions, apoptosis of endothelial cells, proliferation of astrocytes, overactivation of MMP9 and degradation of Col-IV in basement membranes. Abbreviations: IPAD: intramural periarterial drainage; MMP9: matrix metalloprotein 9; PVS: perivascular space; SAH: subarachnoid hemorrhage

In this study, the pattern of clearance of the tracers in ISF within brain parenchyma has been explored normally and after SAH. The results confirmed that solutes in ISF are cleared out of brain, at least partly, via IPAD pathways, with individual characteristics and reach deep cervical lymph nodes. SAH resulted in an impairment of IPAD and of the ECS dynamics, closely associated with capillary injury, reactive astrocytes, upregulation of MMP9 and degradation of Col-IV (Fig. 11).

Convective influx (glymphatic entry) of CSF into the brain is an important factor for brain homeostasis [8]. In this study, firstly, we found that intracisternally injected EB, FITC-D2000 and A-594 were distributed in small vessel PVS in the frontal cortex regardless of their molecular weight, indicating that CSF entered the ECS through astrocyte-dependent PVS pathway. This result confirmed the function of glymphatic system as reported previously [2]. Our results indicated that tracers then entered IPAD pathways to drain to the cervical lymph nodes. Recently, the conventional lymphatic vessels had been found in the dura mater, which was also considered as a pathway for draining ISF [21]. However, the functional relationship between the IPAD system and the lymphatic vessels in dura mater is unclear and still needs further investigation.

We observed the presence of EB in the cervical lymphatic nodes within 0.5 h after injection EB into cisterna magna, confirming previous studies [10]. Blocking the outflow pathway by ligation of dcLNs resulted in the impairment of IPAD. Approximately 15% of tracer from the brain parenchyma draining along the IPAD pass into the CSF and the remaining 85% of the tracer travels along the walls of capillaries to the cervical lymph nodes [12]. The IPAD pathway is not competent for the transfer of antigen presenting cells or lymphocytes, contributing thus to the unique immunological privilege microenvironment within the brain [8].

Our results indicated that lower molecular weight tracers (e.g. A-594) were cleared out of brain via the IPAD pathway, whereas higher molecular weight (e.g. FITC-D2000) did not enter IPAD pathways. This difference may be related to the restricted properties of basement membranes in the arterial tunica media. We cannot speculate if high molecular weight tracers leave the brain, but this may explain the high concentration of amyloid-beta oligomers and fibrils that are unable to clear in neurodegenerative diseases.

After SAH, the clearance of tracers from the brain parenchyma along IPAD decreased, and the normal structure of the IPAD pathway was damaged, leading to the obstruction of ISF drainage. Due to its impermeability to cellular membrane, the contrast Gd-DTPA had been used as an ideal indicator for tracing CSF and ISF flow in brain [14]. Compared with other methods, magnetic resonance imaging (MRI) is able to explore the ISF drainage in deep brain areas such as the hippocampus region [22]. The time profile of the MRI tracer Gd-DTPA allows also the calculation of the ISF drainage parameters, such as diffusion coefficient, clearance coefficient and half-life [22]. Our MRI results showed that, after SAH, there was an evident ISF drainage dysfunction, which revealed indirectly the impairment of IPAD.

The clearance routes of the harmful factors in CSF such as blood cell lysates is still unclear following SAH. In this study, the fluorescent tracers with various molecular weights injected into the in CSF entered brain along perivascular pathways as demonstrated previously, suggesting that toxic substances generated by SAH are able to enter the parenchyma along the same routes and lead to stenosis or vasospasm. Noticeably, the PVS in the frontal cortex was markedly expanded after SAH, associated with the breakdown of the direct junctions between ECS and basement membrane of capillaries, the proximal point for IPAD. A similar pattern has been reported in cerebral amyloid angiopathy [5, 34].

BBB disruption is one a typical consequence after SAH. The BBB is composed of endothelial cells, tight junctions, basement membranes and astrocyte endfeet [15]. Therefore, the apoptosis of endothelial cells and astrocytes, observed in this study, would lead to BBB disruption, vasogenic edema and infiltration of macromolecules such as IgG into the brain from plasma. Since endothelial cells and astrocytes secrete basement membranes which are structural components for IPAD pathways, their apoptosis most likely will have affected the IPAD system.

In this study, we found that the expression and activity of MMP9 increased after SAH. MMP9 is a peptidase enzyme responsible for the degradation of extracellular matrix, contributing to the damage of basement membranes [23]. Previous studies showed that MMP9 was upregulated and played an important role in the pathologic processes during focal cerebral ischemia, and pharmacologic inhibition of MMPs ameliorated edema after focal cerebral ischemia [17]. Activated MMP9 degrades basement membranes, and results in brain edema and hemorrhagic transformation after focal cerebral ischemic episode [26, 33, 36]. We also found that the Col-IV expression level in the basement membrane was accordingly decreased after SAH. Col-IV is the main component of the vascular basement membranes and therefore disruptions of Col-IV will impact upon the integrity of basement membrane and lead to functional impairment [28]. MMP9, the most abundant of MMPs, acts upon collagen, fiber fibrinogen, glass laminin and entactin [4]. Disruptions in Col-IV.

Since SAH is often fatal, the early stage of SAH is mainly life-saving. After the acute stage, more clinical attention is paid towards preventing recurrence and sequelae. Acute and chronic processes should also be considered for a new complete picture of pathophysiological processes. Various pathological processes in the acute stage are relatively intense and some pathological changes are easy to change significantly with time, but the chronic stage is relatively stable, which also provides a new perspective for the evaluation of the clinical significance of this pathological process. In this study, we only explored the IPAD impairment at the acute stage following SAH (24 h), whereas the changes in IPAD and its potential mechanism in chronic processes(> 24 h), potentially different from the acute stage has not been studied. As the failure of IPAD results in cerebral amyloid angiopathy (CAA) [26, 33, 36], the study of IPAD in the chronic stages after SAH may be more clinically significant for the long term complications following SAH, such as CAA.

## Conclusions

Our results showed that, after SAH, there was significant impairment in IPAD system, the perivascular space was markedly expanded and the ISF clearance rate was significantly decreased, associated with the apoptosis of endothelial cells, activation of astrocytes, over-expression of MMP9 and loss of Col-IV.

## Data Availability

The datasets used and/or analysed during the current study available from the corresponding author on reasonable request.

## Abbreviations

AD: Zheimer’s disease
AQP4: Aporin-4
BBB: Brain-barrier
BSA: Bovine serum albumin
CAA: Cerebral amyloid angiopathy
Col-IV: Collagen type IV
CSF: Cerebrospinal fluid
CVBM: Capillary vessel basement membrane
dcLN: Deep cervical lymph node
EB: Evans Blue
ECS: Extracellular space
Gd-DTPA: Gadolinium-diethylenetriaminepentaacetic acid
GFAP: Glial fibrillary acidic protein
IPAD: Intramural periarterial drainage
ISF: Interstitial fluid
ISS: Interstitial space
LSCM: Laser scanning confocal microscopy
MMPs: Matrix metalloproteinases
MMP9: Matrix metalloproteinase 9
MRI: Magnetic resonance imaging
PVS: Perivascular spaces
SAH: Subarachnoid haemorrhage
scLNs: Superficial cervical lymph nodes
SD: Standard deviation
TEM: Transmission electron microscopy
TPF: Two-photon fluorescent
TUNEL: TdT-mediated dUTP nick-end labeling
